# Electrically-pumped compact topological bulk lasers driven by band-inverted bound states in the continuum

**DOI:** 10.1038/s41377-023-01200-8

**Published:** 2023-06-12

**Authors:** Song Han, Jieyuan Cui, Yunda Chua, Yongquan Zeng, Liangxing Hu, Mingjin Dai, Fakun Wang, Fangyuan Sun, Song Zhu, Lianhe Li, Alexander Giles Davies, Edmund Harold Linfield, Chuan Seng Tan, Yuri Kivshar, Qi Jie Wang

**Affiliations:** 1 Centre for Optoelectronics and Biophotonics, School of Electrical and Electronic Engineering & The Photonics Institute, Nanyang Technological University, Singapore, Singapore; 2 Electronic Information School, Wuhan University, Wuhan, China; 3 School of Electronic and Electrical Engineering, University of Leeds, Leeds, UK; 4 Nonlinear Physics Center, Research School of Physics, Australian National University, Canberra, ACT 2601 Australia; 5 Division of Physics and Applied Physics, School of Physical and Mathematical Sciences, Nanyang Technological University, Singapore, Singapore

**Keywords:** Quantum cascade lasers, Terahertz optics

## Abstract

One of the most exciting breakthroughs in physics is the concept of topology that was recently introduced to photonics, achieving robust functionalities, as manifested in the recently demonstrated topological lasers. However, so far almost all attention was focused on lasing from topological edge states. Bulk bands that reflect the topological bulk-edge correspondence have been largely missed. Here, we demonstrate an electrically pumped topological bulk quantum cascade laser (QCL) operating in the terahertz (THz) frequency range. In addition to the band-inversion induced in-plane reflection due to topological nontrivial cavity surrounded by a trivial domain, we further illustrate the band edges of such topological bulk lasers are recognized as the bound states in the continuum (BICs) due to their nonradiative characteristics and robust topological polarization charges in the momentum space. Therefore, the lasing modes show both in-plane and out-of-plane tight confinements in a compact laser cavity (lateral size ~3λ_laser_). Experimentally, we realize a miniaturized THz QCL that shows single-mode lasing with a side-mode suppression ratio (SMSR) around 20 dB. We also observe a cylindrical vector beam for the far-field emission, which is evidence for topological bulk BIC lasers. Our demonstration on miniaturization of single-mode beam-engineered THz lasers is promising for many applications including imaging, sensing, and communications.

## Abstract (web summary)

Topological bulk BICs in the vicinity of inverted photonic band edges enable an electrically pumped, compact, single-mode, and beam engineered QCL to be operated at terahertz region.

## Graphical abstract

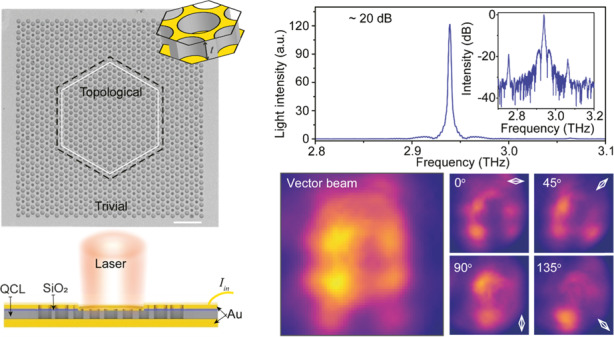

## Introduction

Topological phase transition classifies the nontrivial bulk band topology of electronic materials^1^. While being insulator in the bulk, the surface of topological insulator supports conducting states that are immune to disorders and/or defects. The introduction of topological phase transition to the design of photonics has led to the advent of photonic topological insulator (PTI)^2–4^. Due to their flexible building blocks and size-dependant frequency responses, the PTIs can be tailored by mimicking diverse topological phenomena, including quantum Hall effect^5–10^, quantum spin-Hall effect (QSHE)^11–14^, quantum valley Hall effect (QVHE)^15–18^, high-order topological insulator (HOTI)^19–23^, and three-dimensional (3D) topological insulator^24–26^, ranging from the acoustics and the microwave to the visible wavelengths. In recent years, considerable efforts have been devoted to applying the PTIs for real-world applications, such as optical delay lines^27^, THz communications^28^, and lasers^29–52^. Among these advanced applications, topological insulator lasers (TILs) attract great attentions^29–52^. In TILs, lasing modes in a topological cavity are amplified by the gain medium and coupled to the continuum of free space through certain emission channels as laser radiation. For lasing modes that are below the light line, they are usually less radiative. A typical method to overcome such problem is to employ grating couplers or on-site defects for enhancing the light outcoupling^30,35,42,46,49^. In comparison, some other topological states within the light cone, e.g., in the QSHE cavity that inherently couple to the radiation continuum, are available for topological edge states probe by spectroscopic means as well as high-performance lasers^52,53^. In ref. ^52^, the band-inversion induced reflection mechanism achieved lasing from the orbital $l=0$ mode in an optical pumping setup with objective lens with angle of convergence for collimation of pump beam and collection of emission beam. Such orbital $l=0$ mode exists at a specific in-plane *k* in the momentum space, thereby making it a regular band-inversed leaky mode.

The coupling of photonic leaky modes to the continuum of free space usually manifests the Fano line-shape^53^. Particularly, the vanished linewidth of photonic leaky modes above the light line gives rise to a new physical mechanism for extreme light localizations, which is known as the bound states in the continuum (BICs)^54^. Photonic BIC modes, describing the radiative characteristics of *k*-space band structure, are of great interest for light confinement to a 2D photonic crystal slab. The in-plane light confinement usually requests a large-area design^55,56^, or a discontinuous reflection barrier to enhance mode confinement^57,58^. However, for the nontrivial and trivial topological phase transitions of the bulk bands, they support a naturally induced band-inversion reflection. As a consequence, the photonic BIC and the band-inversion induced reflection can be combined together to significantly promote the cavity designs for exploring the fundamental limit of miniaturizing topological laser cavity and finally contribute to integrated optoelectronic devices.

The electrically pumped semiconductor lasers are among the most important sources owing to their high efficiency, compactness, and solid-state stability^59,60^. For the mid-infrared and terahertz radiations, quantum cascade lasers (QCLs) are the most important sources operating under electrical pumping^61,62^, which have been implemented by isolated ultracompact resonators^63,64^. While demonstrating single-mode lasing, the emission power from such isolated cavity is usually weak. To enhance the emission power, artificially designed distributed-feedback (DFB) gratings can be patterned on the top of a ridge laser^65–68^. As results, the DFB-based QCLs can give rise to near Watt-level emission and maintaining single-mode emission at the same time due to their interfered diffraction mechanism for mode selection and amplification^67,68^. However, in addition to single-mode operation, research on QCLs still request for nontrivial beam engineering (e.g., circular polarized beams, vortex beams, and vector beams) and high optical emission from a single laser device^56,69^, which is highly desired for advanced optoelectronic applications, such as communications, imaging, spectroscopy, and so on.

Here, we report an electrically pumped compact topological bulk laser that is governed by the mechanism of BICs. We demonstrate that the band-inversed quadrupolar band edges show infinite Q-factors in the centre of the Brillouin zone, known as the symmetry-protected BICs, under periodic boundary conditions. We further calculate the far-field polarization windings, showing topological charge of +2, which is the essential criteria for recognizing photonic BICs. By constructing two domains with a nontrivial domain surrounded by a trivial domain, we show that pure BIC states degrade into quasi-states due to the finite topological bulk cavity size. In addition to the band-inversion that can introduce perfect reflection at the topological interface, we further show that the demonstrated topological bulk quasi-BICs can always support higher Q-factors as compared to photonic cavities which only have the nontrivial domains. As a result, we realize an efficient single-mode (SMSR ~ 20 dB) and beam engineered QCL operating under electrical pumping and with a compact footprint (laser cavity size ~3λ_laser_) even the gain spectrum of such QCL wafer is broadband.

## Results

### Topological band inversion and BICs at the quadrupolar band-edges

**Fig. 1:**
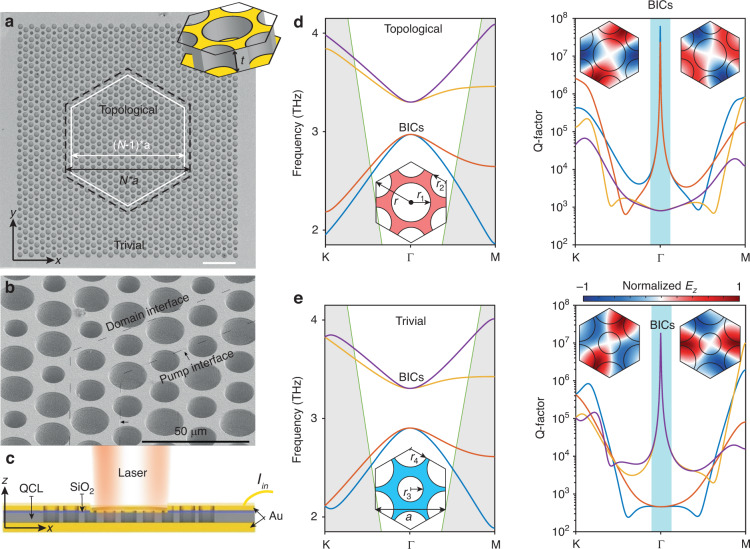
Fabricated sample and bound state in the continuum (BIC) features of the quadrupolar band edges. **a**, **b** The scanning electron microscope (SEM) image of the fabricated laser cavity, where the pump region with lateral periodic number of *N*-1 is the topological nontrivial domain encircled by the dashed white hexagon box. The actual interface between the trivial and nontrivial domains is indicated by the black box, where the topological bulk cavity has lateral periodic number of *N*. The scale bar in (**a**) indicates 100 μm. The inset show the 3D perspective view of the designed photonic supercell with QCL thickness of 13 μm. **c** The cut-view (schematic) of the laser device. **d**, **e** Band structures and Q-factors of the topological and nontrivial supercells under periodic boundary conditions. The gray shadows show the region that is below the light line. The supercell structures for the trivial and nontrivial domains are shown by the insets, respectively. The lattice constant $a=35$ μm, and the radius of the hexagon $r=a/\sqrt{3}$. With fabrication error taken into consideration (see Supplementary Materials for details), the topological and trivial supercells have geometric parameters of ${r}_{1}=0.48316r$, ${r}_{1}=0.38316r$, ${r}_{3}=0.31316r$ and ${r}_{4}=0.45316r$, respectively. The quadrupolar modes (${d}_{{xy}}$ and ${d}_{{x}^{2}-{y}^{2}}$ with the normalized electric fields, z-components, shown by the inset mode profiles) show BIC features as their Q-factors tend to diverge to infinity at the Brillouin zone center (Γ point). The cyan belts show the regions where the quadrupolar modes appear as BICs and they have inversed frequencies for the topological case

The proposed topological cavity consists of two domains with the topological domain surrounded by the trivial domain, both coordinated in a two-dimensional (2D) hexagonal lattice with the lattice constant a = 35 μm, which is etched as cylindrical holes through the QCL wafer with thickness (*t*) of 13 μm, as shown in Fig. 1a. The QCL wafer is cladded by double metals (Ti/Au stack in our case), but only the pumped region, i.e., the topological bulk cavity, is connected directly with the top metal layer and the other region is isolated by an insulating SiO_2_ layer with a thickness of 250 nm, as shown in Fig. 1c. The fabrication details can be found in refs. ^42,69^. The band structures are TM-polarized due to such double-metal claddings. To avoid interactions between the topological bulk states and the edge states due to pump current spreading, the actual pump region is reduced by about one period with a reference to the physical interface between the non-trivial and the trivial regions, as shown in Fig. 1a, b, where the actual pump region is shown inside the white hexagon box, and the black dashed hexagon indicates the topological interface. The supercells for both the topological and trivial designs are shown in Fig. 1d, e, respectively. The topological supercell has an etched cylindrical air hole with radius of r_1_ in the middle and six etched cylindrical air holes at the hexagon apexes with radius of r_2_, as shown in Fig. 1d. Here, r_1_ is larger than r_2_, indicating a larger intra-cell coupling than the inter-cell coupling (see Supplementary Information for details). Therefore, the corresponding band structure has a band inversion with the quadrupolar modes transiting to the lower frequency, while the dipolar modes appear at the higher frequencies, as shown on the left panel of Fig. 1d. The electromagnetic field with the normalized *E*_*z*_-component (TM-polarization) are confined inside the QCL wafer (connection bridges between Au claddings), which gives rise to the inter-/intra-cell mode couplings, i.e., the connection bridge becomes thinner which can squeeze the mode volume of *E*_*z*_-field to make the coupling stronger, and vice versa (see the inset figures in the right panels of Fig. 1d, e and also in Fig. S2 in the Supplementary Information). To our interests, we pay special attention to the Q-factors of these bands and find that two degenerated quadrupolar modes have infinite Q-factors at the Γ-point. Therefore, they are symmetry-protected BICs as these states are intrinsically above the light line^54^. In comparison, the trivial supercell has an etched cylindrical air hole with radius of r_3_ in the middle and six etched cylindrical air holes at the hexagon apexes with radius of r_4_ with the relation r_3_ < r_4_, as shown in Fig. 1e. The intra-cell coupling is therefore smaller than the inter-cell coupling, and a trivial bandgap opens where the quadrupolar modes also show infinite Q-factors, i.e., BIC modes, at the Γ-point. We then construct a domain wall where the topological domain is surrounded by the trivial domain. If there is an optical mode that only exists in the topological domain, its propagation to the trivial domain in the lateral direction must cross the topological interface into the trivial domain which has a distinct topological phase. As the topological bulk BIC mode presented in this work has the highest Q-factor cavity mode, it becomes the dominating lasing mode. In addition to the band-inversion-induced reflection for mode confinement in the topological (the pumping) region, the BIC mechanism provides another degree of freedom to further confine the optical lasing mode even at a finite cavity size^55^. Therefore, it is a highly desired design for miniaturized laser devices.

**Fig. 2:**
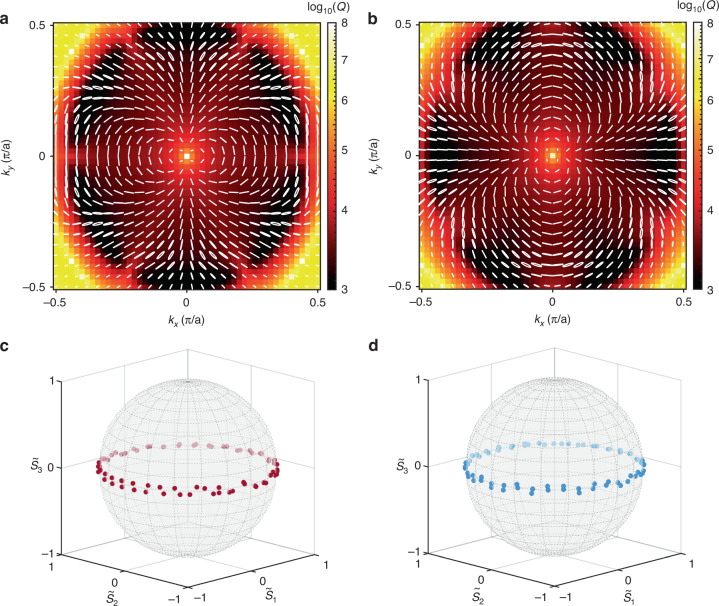
Polarization topologies of the BICs at the vicinity of the quadrupolar modes. **a**, **b** The far-field polarizations of the topological supercell. The charge number of the polarization winding is +2. **c**, **d** The projected polarization states at the vicinity of the quadrupolar modes are illustrated by the normalized Stokes parameters on the Poincare sphere

To further understand the degenerated quadrupolar modes as BICs, we calculated their Q-factors and polarization states of the outgoing plane waves in the 2D momentum space, as shown Fig. 2a, b. Here only the two degenerated quadrupolar modes of the topological supercell are analyzed. The analyses for trivial supercell are included in the Supplementary Materials. The far-field topological features of BICs are also revealed by the polarization vortices that are winding around the diverged Q-factors. The topological charge (*q*) of the polarization vortex is therefore calculated as $q=\frac{1}{2\pi }\mathop{\oint }\limits_{C}d{\boldsymbol{k}}\,{\cdot }\,{\nabla }_{k}\phi \left({\boldsymbol{k}}\right)$, where *C* is a counter-clockwise closed loop surrounding the polarization vortex center, $\phi \left({\boldsymbol{k}}\right)={ang}\left({c}_{x}\left({\boldsymbol{k}}\right)+i{c}_{y}\left({\boldsymbol{k}}\right)\right)$ is the angle of the polarizations at different projected in-plane wavevector ${\boldsymbol{k}}=\left({k}_{x},{k}_{y}\right)$, with the *x* and *y* components denoted by ${c}_{x}\left({\boldsymbol{k}}\right)$ and ${c}_{y}\left({\boldsymbol{k}}\right)$, respectively. The calculated topological charges are +2 that indicates the polarization winds around the BIC by two times. Such polarization topology can also be projected to a Poincare sphere, as shown in Fig. 2c, d, respectively. The polarization states are now described as the normalized Stokes parameters $$\left(\begin{array}{cc}\begin{array}{cc}1 & {\widetilde{S}}_{1}\end{array} & \begin{array}{cc}{\widetilde{S}}_{2} & {\widetilde{S}}_{3}\end{array}\end{array}\right)$$, with ${\widetilde{S}}_{1}={S}_{1}/{S}_{0}$, ${\widetilde{S}}_{2}={S}_{2}/{S}_{0}$, and ${\widetilde{S}}_{3}={S}_{3}/{S}_{0}$, where ${S}_{0}={\left|{c}_{x}\left({\boldsymbol{k}}\right)\right|}^{2}+{\left|{c}_{y}\left({\boldsymbol{k}}\right)\right|}^{2}$, ${S}_{1}={\left|{c}_{x}\left({\boldsymbol{k}}\right)\right|}^{2}-{\left|{c}_{y}\left({\boldsymbol{k}}\right)\right|}^{2}$, ${S}_{2}=2\mathrm{Re}\left[{c}_{x}^{* }\left({\boldsymbol{k}}\right){c}_{y}\left({\boldsymbol{k}}\right)\right]$, and ${S}_{3}=2{Im}\left[{c}_{x}^{* }\left({\boldsymbol{k}}\right){c}_{y}\left({\boldsymbol{k}}\right)\right]$, respectively. For the iso-frequency circle with $\left|{\boldsymbol{k}}\right|=0.05$ that is in the vicinity of the BICs, the polarization states are approximately linear-polarized. Therefore, all these Stokes parameters are projected to the equator of the Poincare sphere. Moreover, these projected Stokes parameters also wind around the equator of the Poincare sphere by two times according to the +2 topological charge of the far-field polarization, as shown by the paired polarization states in Fig. 2c, d.

### Miniaturization of the topological bulk BIC laser cavity

**Fig. 3:**
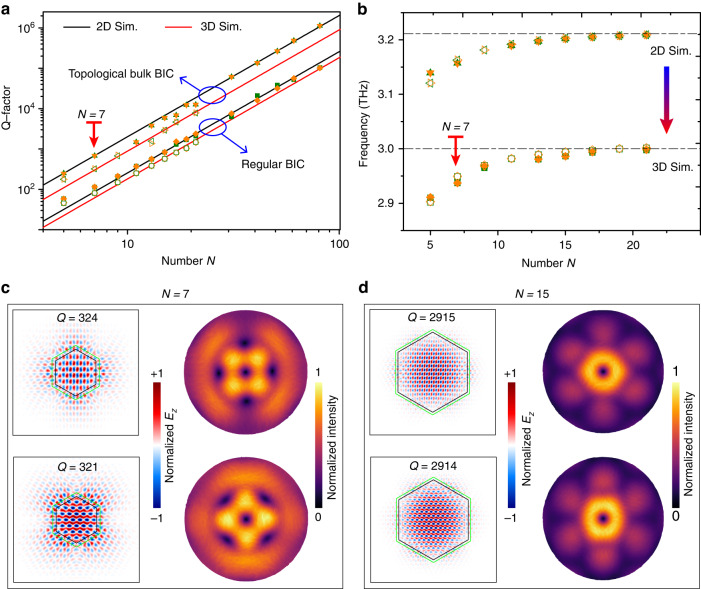
Miniaturized design of the topological bulk BIC laser cavity. **a** Comparison of the passive Q-factors between the topological bulk BIC cavity (two domain) and a regular BIC cavity that only has topological nontrivial domain. **b** Frequency distributions by varying the size of the topological domain. **c** Near-field and far-field profiles of the quasi-BICs for topological bulk cavity with lateral periodic number *N* = 7. The far-field beam shows perturbated cylindrical vector beam with center region demonstrating singularities. **d** Near-field and far-field profiles of the quasi-BICs for topological bulk cavity with lateral periodic number *N* = 15. The far-field beam shows clear cylindrical vector beam. For both devices (*N* = 7 and *N* = 15), the QCL slab is lossy outside the black hexagonal border (imaginary part of the refractive index as 0.016), while it is lossless inside the black hexagonal border. The green hexagonal border indicates the nontrivial-trivial domain interface

In practice, a fabricated laser device always has a finite size. As a consequence, the pure BIC states with degenerated frequencies are transformed into quasi-states, as shown in Fig. 3a. Here, the pumped region within the black hexagon box is lossless, while the outside region is lossy, as shown in Fig. 3c, d, respectively. Figure 3a shows the numerically simulated Q-factors for devices at different lateral periodic number (*N*) in 2D and 3D simulations. For 3D simulations, the BIC lasers show lower Q-factors as compared to 2D simulations, as the radiative loss along the emission direction is taken into consideration in 3D simulations. The two-domain designs (topological bulk BIC) with the domain wall show much higher Q-factors compared to the one-domain designs (regular BIC). Owing to the band-inversion induced reflection, such two-domain design can also approach the true BIC states even at a relatively small cavity size. In other words, the topological bulk BIC design indeed allows the laser cavity to operate at highly compact footprint, which is promising for energy-efficient and monolithically integrated real-world applications. Figure 3b plots the frequencies of the quasi-BICs. Both 2D and 3D simulations show that the BIC frequencies start to saturate and are pinned to the BIC band edges for the topological bulk cavity size with *N* > 15. With the Q-factors and eigenfrequencies of the quasi-BICs presented, we aim to experimentally explore the most compact laser device under electrical pumping.

The near-field (electric field, z-component) and far-field (electric intensity) distributions of two different devices with lateral periodic numbers of *N* = 7 and *N* = 15 are shown in Fig. 3c, d, respectively, where their doubly degenerated modes (i.e., ${d}_{{xy}}$ and ${d}_{{x}^{2}-{y}^{2}}$) are clearly observed, and most of the energy are tightly confined in the topological bulk region. Although operating with a finite size, the quasi-BICs can generate a vector-like beam that exhibits doughnut-like profiles in the far-field^54–57^. To demonstrate such a phenomenon, we set an observation plane (*z* > 500 μm) above the topological bulk cavity, see Supplementary Fig. S6 for details. The observed far-field patterns fit well with the theoretical demonstrations. For the sample with the lateral topological bulk cavity with *N* = 7, the outer region of the beam doesn’t show circular shape which is mainly due to the penetrated near-field energy from the topological domain to the trivial domain. The energy penetration in the trivial domain is gradually decayed. The topological bulk cavity size, for example *N* = 15 shown in Fig. 3d, can be enlarged to avoid the lateral energy penetration from the topological domain to the trivial domain, thereby enhancing the purity of the emitted beam profile. It is worth mentioning that the topological bulk BIC cavity can also be fabricated with a large dimension to enhance the emitted laser power for practical applications^51,56,69^, even though the work presented here focuses on a compact size laser cavity.

### Characterization of the topological bulk BIC laser

**Fig. 4:**
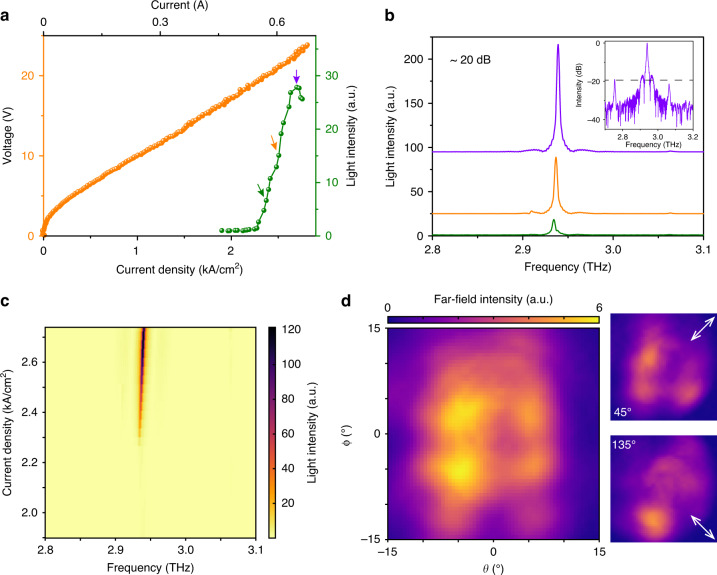
Experimental measured topological bulk BIC laser. **a** Measured light-current-voltage (L-I-V) curve. **b** The laser spectra at different pump current density as function of frequency. The spectrum with maximum emission power is employed for the side-mode suppression ratio (SMSR) calculation. The measured SMSR is around 20 dB. **c** 2D mapping of the lasing spectra as functions frequency and pump current density. **d** The measured far-field beam profile using a Golay cell detector. A polarizer was inserted and rotated in front of the detector to resolve the beam profiles, where two lobes are distinguished for 45° and 135°, respectively

The laser emission from the fabricated device was measured using a customized experimental setup, where the laser chip was mounted in a cryostat with the operation temperature stabilized at 8.5 K^42,69^. An electrical pulse generator was employed to pump the topological bulk BIC laser in pulsed mode operation with a repetition rate of 10 kHz and a pulse width of 500 ns, which gives rise to a duty cycle of 0.5%. If the laser is operated in continuous wave mode, normally such 2D laser cavities will have an impact on the highest temperature operation due to the thermal dissipation effects. The emitted laser spectra were measured by a Fourier transform infrared spectrometer (FTIR, Bruker Vertex 80 series) with a spectral resolution of 0.08 cm^−1^. The experimental light-current-voltage (L-I-V) curves of a sample with lateral periodic number of 7 are plotted in Fig. 4a, where the lasing threshold is around 2.2 kA cm^−2^ and rollover is around 2.71 kA cm^−2^ (dynamic range of 0.5 kA cm^−2^). The laser spectra are shown in Fig. 4b, c. With increasing pump, single-mode laser emission can be clearly identified whose frequency is at around 2.93 THz, very close to the predicted BIC frequency through simulations. A slight blueshift of the laser peak is observed as the applied electric field generates Stark shifts of the intersubband transition in the THz QCL medium. The single-mode laser performance is evaluated by the calculated side-mode suppression ratio (SMSR) when the laser output power is maximum, as shown in Fig. 4b. The SMSR can reach 20 dB for the topological bulk BIC laser device under investigation.

To visualize the emitted beam profile, a homemade 2D scanning setup was employed to scan far field, where the alignment process and measurement details can be found in ref. ^69^. A THz Golay cell detector, model of TYDEX GC-1T with aperture diameter of 11 mm, was mounted on the 2D mechanical stage to scan the laser beam profile. To improve the signal-to-noise ratio, the topological bulk BIC laser was driven by a pump current with repetition rate of 10 kHz and pulse width of 1000 ns (duty cycle of 1%), and another 15 Hz electrical modulation was further imposed for lock-in amplification of the detector signal. An iris with an aperture diameter of 4 mm was inserted between the QCL and the detector to narrow down the scanning pixel size, and thus improve the far-field resolution. The measured QCL beam is shown in Fig. 4d, which is a doughnut-like cylindrical vector (CV) beam with a beam divergence of about 10°. This divergence is also confirmed by a 3D far-field simulation, which is included in the Supplementary Materials. To confirm the CV characterization of the far-field beam, a polarizer was inserted and rotated at front of the detector to resolve the beam profiles. For the polarization angles of 45° and 135°, two lobes in the far field are distinguished correspondingly, which further confirm the emitted laser as a CV beam. The polarization-resolved beams from 0° to 135° are compared in the Supplementary Materials (Figs. S8 and S9). Reasonably good agreements can be identified between the experiments and numerical simulations. It should also be mentioned that only one topological bulk BIC mode is lasing that is due to the gain competition. Therefore, the measured beam profile also fits well with the topological bulk BIC mode with higher Q-factor that is shown by the up panel of Fig. 3c.

## Discussion

We have implemented an electrical pumped topological bulk BIC laser that shows single-mode operation (SMSR around 20 dB) and the cylindrical vector beam-like emission in the THz frequency region. The topological bulk BIC engages both vertical (out-of-plane) and lateral (in-plane) confinement that enhances coherent emission. In addition, the topological band inversion-induced reflection further strengthens the lateral confinement for the topological bulk BIC mode. As a result, we realize a miniaturized and single-mode laser device even its lateral cavity dimension is only 3λ_BIC_. We believe such electrical pumped BIC THz QCL is promising for energy efficiency and monolithic integration, making it highly attractive for THz integrated electronic and photonic applications.

## Materials and methods

### Materials and device fabrication

The employed QCL wafer in this work has a gain curve spans from 2.7 THz to 3.2 THz, verified by the emission spectrum envelope of a ridge laser fabricated on the same wafer (see Supplementary Fig. 1 in detail). The device fabrication follows the standard process for metal-semiconductor-metal configuration that is given by ref. ^69^.

### Numerical simulations

The COMSOL Multiphysics was employed to numerically simulate 3D full-wave results. To simulate the band structure, the QCL, with active region thickness of 13 μm and refractive index of 3.85, was set as a lossless and dispersion free medium. The PEC metal with configuration given by the inset in Fig. 1a was employed to cover the QCL. For the cavity simulation, the pump region is lossless and dispersion-free with parameters same as the supercell, while the unpumped region has an imaginary part of 0.016 for the refractive index. In addition, two gold layers, modelled as lossy metal with refractive index of 182.67 + 212.11i, with thickness of 300 nm form the top and bottom contacts.

## Supplementary information

Supplementary Materials

## Data Availability

All data and related codes in the paper are present in the main text and/or the Supplementary Materials, which will also be provided from the corresponding author upon reasonable request.
