# Supplementary material for: Electrically-pumped compact topological bulk lasers driven by band-inverted bound states in the continuum
Source: Light Sci Appl. 2023 Jun 12;12:145. doi: 10.1038/s41377-023-01200-8 (PMC10261106; doi:10.1038/s41377-023-01200-8)
Supplement: Supplementary file 1 — Supplementary Materials [file 41377_2023_1200_MOESM1_ESM.docx]

**Supplementary Materials for “Electrically-pumped compact topological bulk laser driven by band-inverted bound states in the continuum”**

Song Han^1,^ ^6,^ *, Jieyuan Cui^1, 6^, Yunda Chua^1, 6^, Yoqngquan Zeng^2^, Liangxing Hu^1^, Mingjin Dai^1^, Fakun Wang^1^, Fangyuan Sun^1^, Song Zhu^1^, Lianhe Li^3^, Alexander Giles Davies^3^, Edmund Harold Linfield^3^, Chuan Seng Tan^1^, Yuri Kivshar^4^, Qi Jie Wang^1. 5,^ *

^1^Centre for Optoelectronics and Biophotonics, School of Electrical and Electronic Engineering & The Photonics Institute, Nanyang Technological University, Singapore.

^2^Electronic Information School, Wuhan University, Wuhan, China.

^3^School of Electronic and Electrical Engineering, University of Leeds, Leeds, UK.

^4^Nonlinear Physics Center, Research School of Physics, Australian National University, Canberra ACT 2601, Australia.

^5^Division of Physics and Applied Physics, School of Physical and Mathematical Sciences, Nanyang Technological University, Singapore.

^6^These authors contributed equally to this work.

*Correspondence to: Dr. Song Han ([song.han@zju.edu.cn](mailto:song.han@zju.edu.cn)) or Prof. Qi Jie Wang ([qjwang@ntu.edu.sg](mailto:qjwang@ntu.edu.sg))


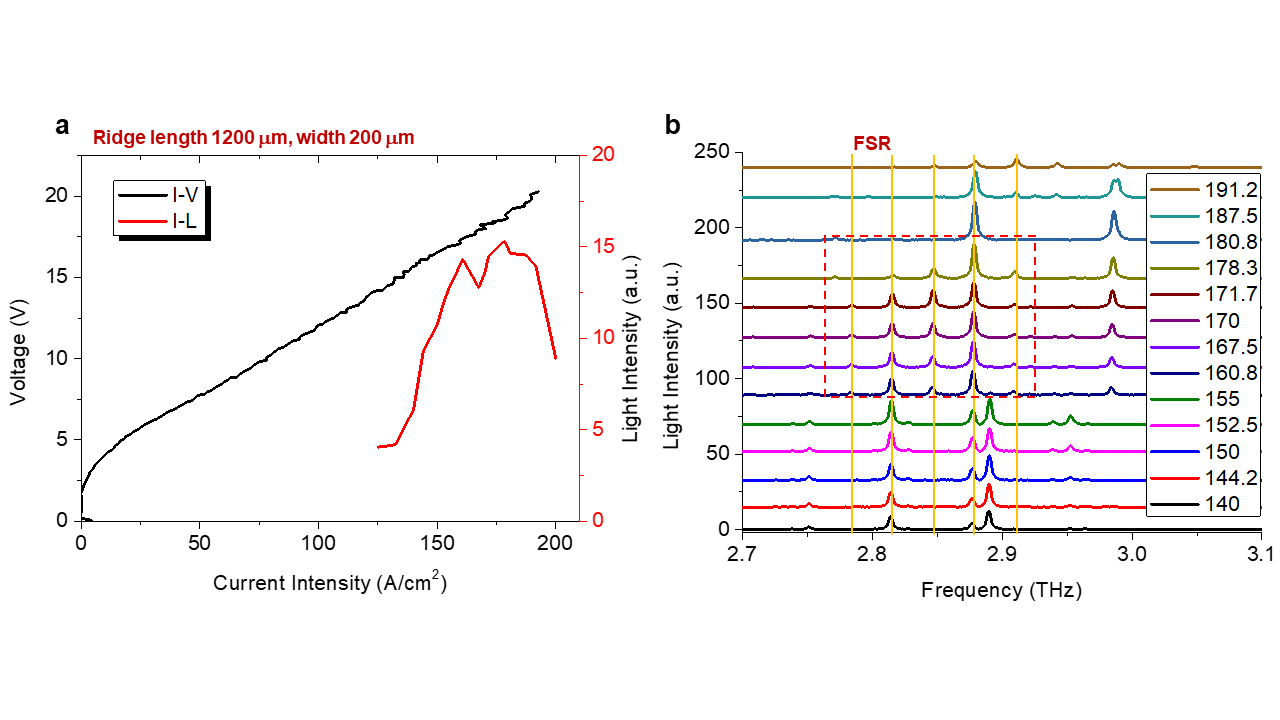


**Figure S1**. Conventional ridge laser has been fabricated and characterized. **a**, The L-I-V cures and emission spectra of a ridge laser at pump current densities scanning through the entire dynamic range. **b**, The gain spectral range is approximately from 2.7 THz to 3.1 THz. The free spectra range (FSR) is employed to estimate the effective refractive index (n) of the QCL under pump. For the current wafer, the calculated n = 3.82.


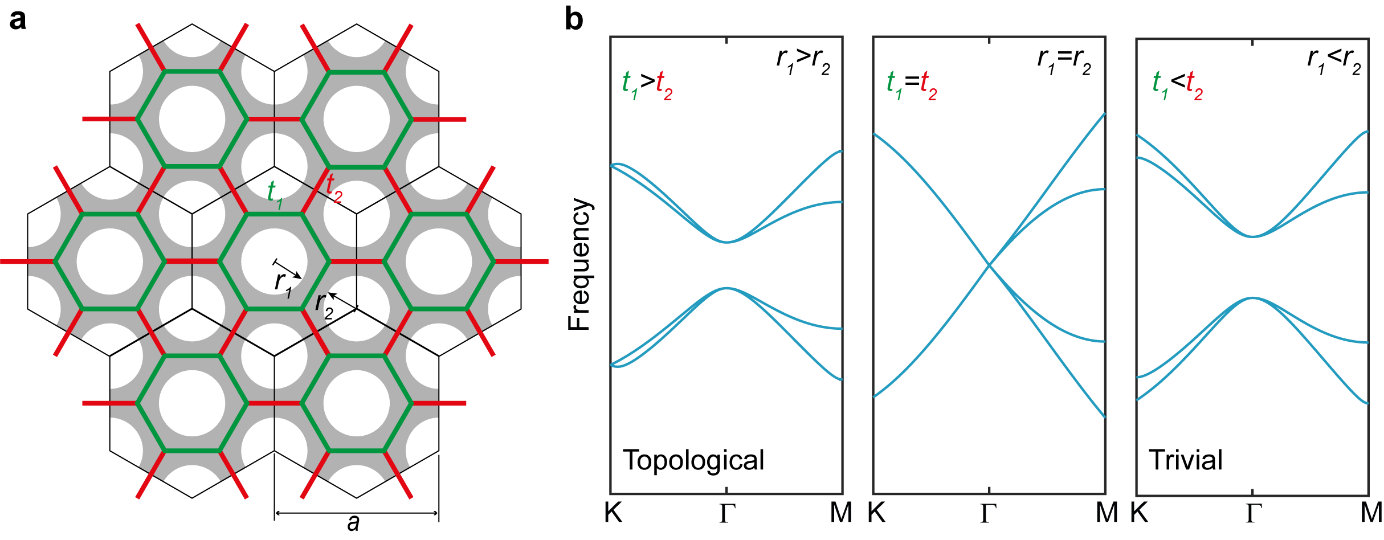


**Figure S2**. Tight-binding model (TBM) and band structures for distinguishing the topological and trivial phases. **a**, the green connections indicate the intra-supercell coupling and the red connections imply the inter-supercell coupling, respectively. The gray background shows the QCL wafer that cladded by double metal (Au) layers. The white circles indicate the drilled air holes inside the QCL wafer. **b**, the TBM-calculated band structures for the topological phase (band inversion), Dirac cone, and the trivial phase, which have one to one correspondence to the manipulation of the radius of the drilled air holes (showing in the main text).


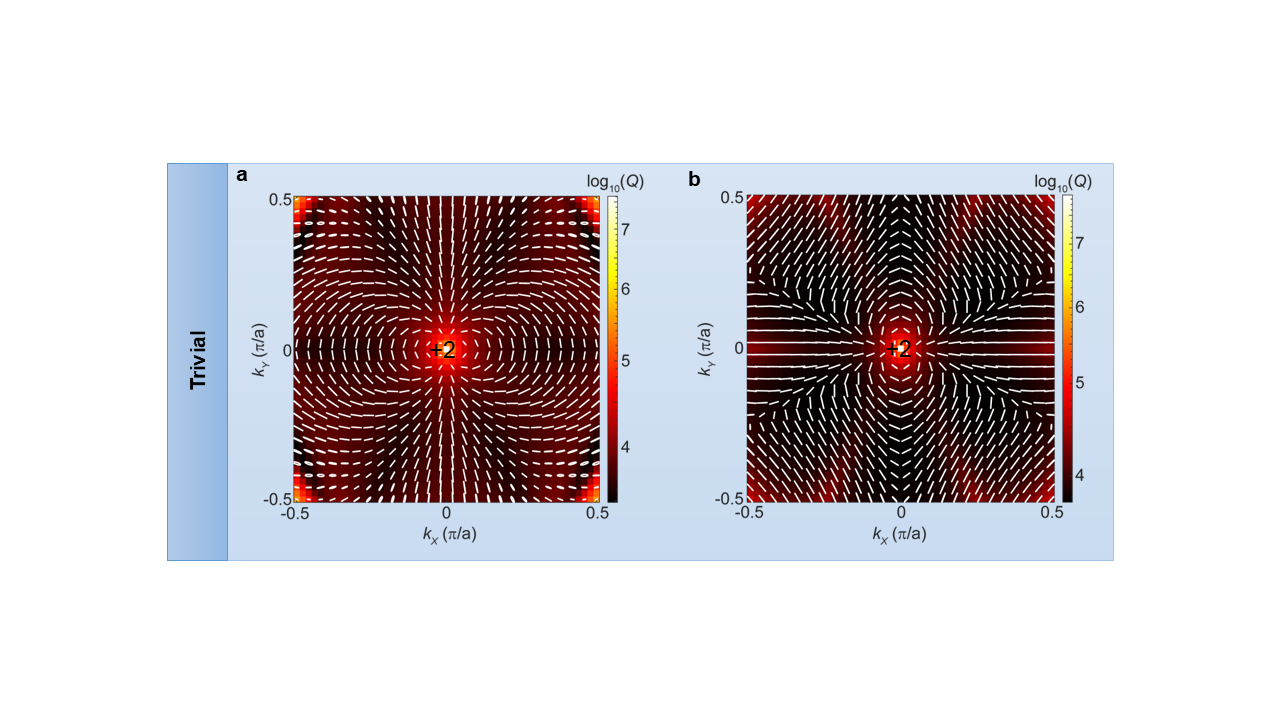


**Figure S3**. The polarization topologies of the BICs on the degenerated quadrupolar bands of the trivial lattice. The charge number of the polarization winding is +2.


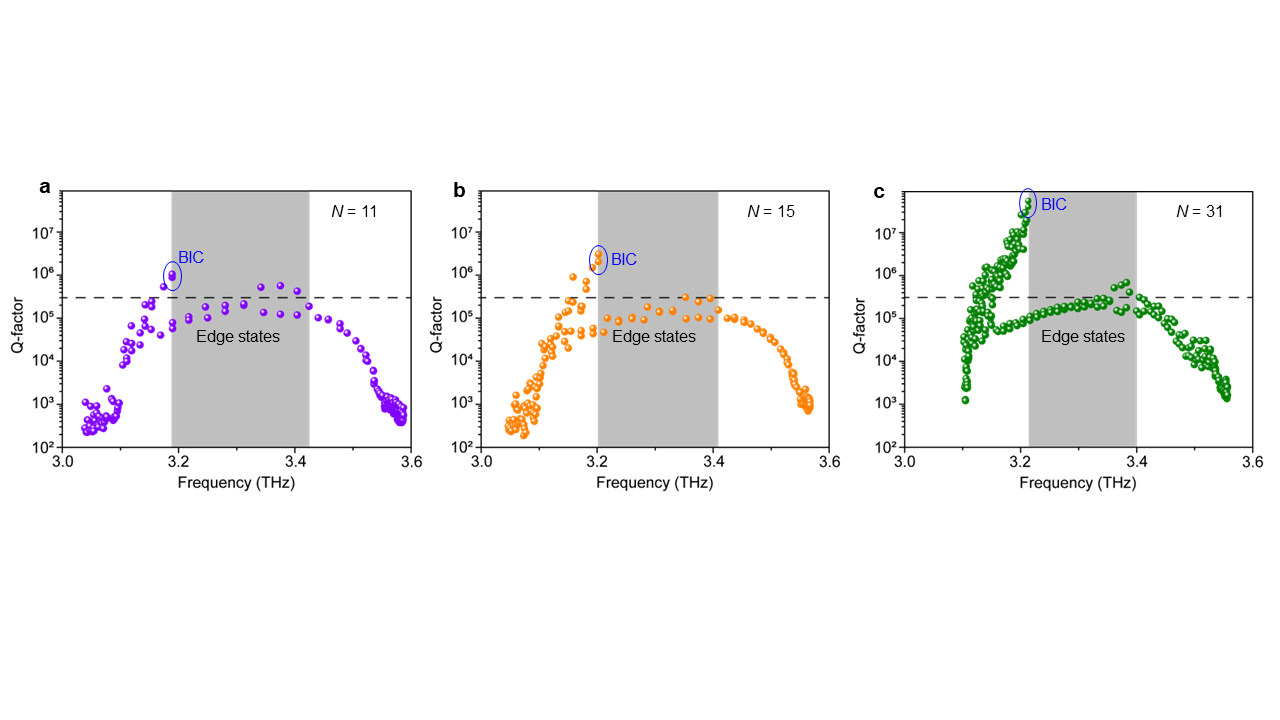


**Figure S4**. 2D simulations for the topological domain surrounded by the trivial domain, both domains are lossless. As results, the edge states and bulk states are observed at their maximum value. The lateral side length (*L*) of the topological domain is **a**, *N* = 11; **b**, *N* =15; and **c**, *N* = 31, respectively. The Q-factor of topological edge states are almost unchanged for different structures, while the band edges (i.e., the BICs) are monotonously enhanced. This implies that only the BICs can give rise to high-power emission as their Q-factor can be manipulated by simply increasing the cavity size.


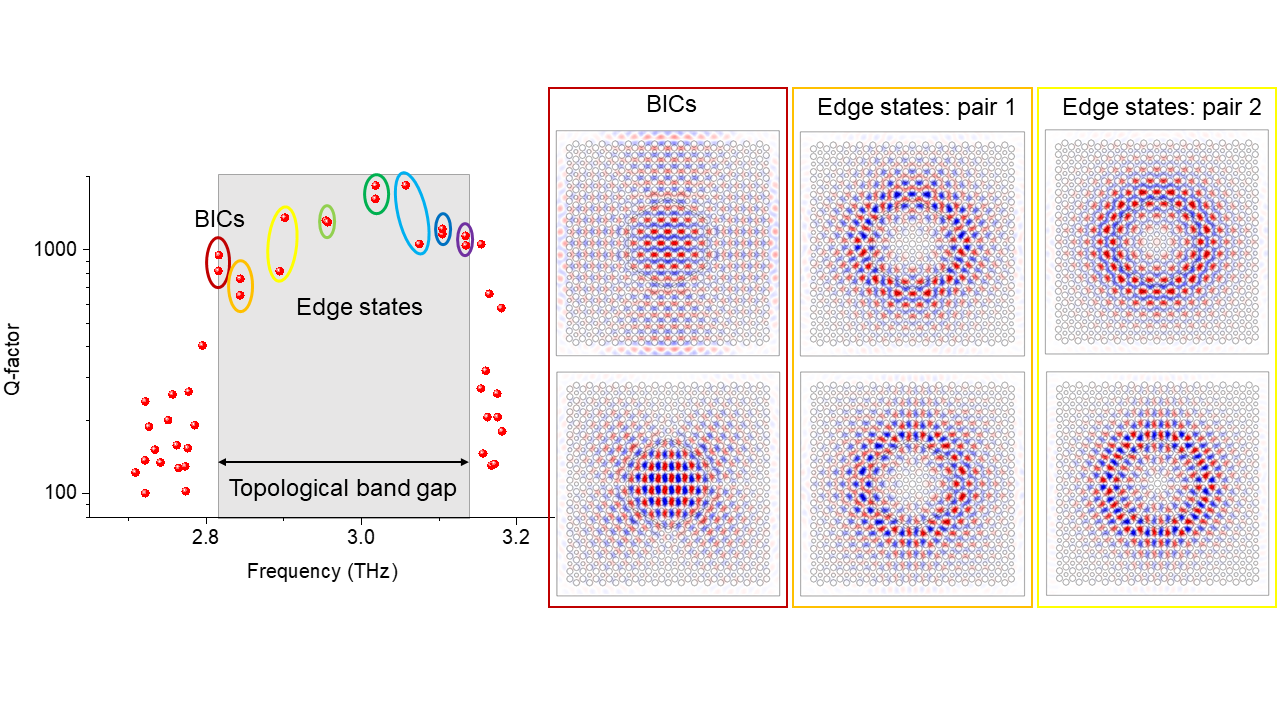


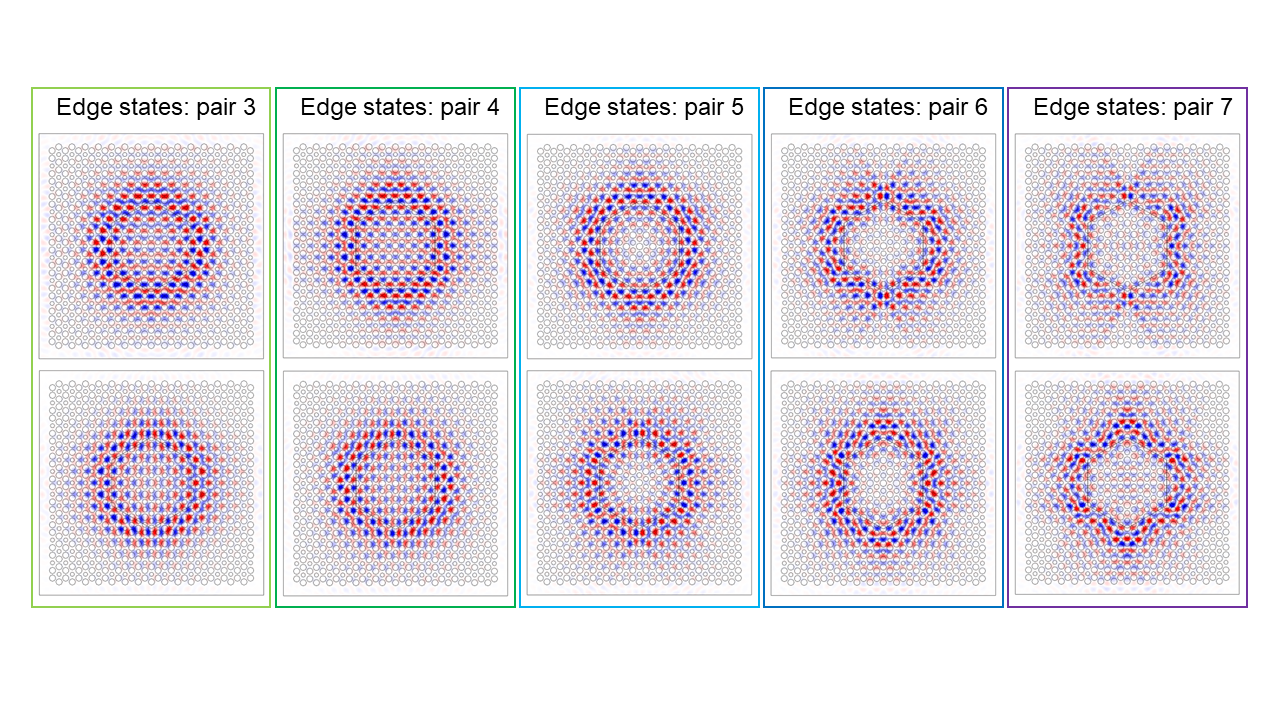


**Figure S5**. 3D simulated Q-factors and frequency distributions of the topological bulk BIC modes and topological edge states for a lossless device (N = 7). The corresponding near-field distributions are also listed. The gray shadow indicates the topological bandgap. It should also be noted that here the fabrication error isn’t taken into account.


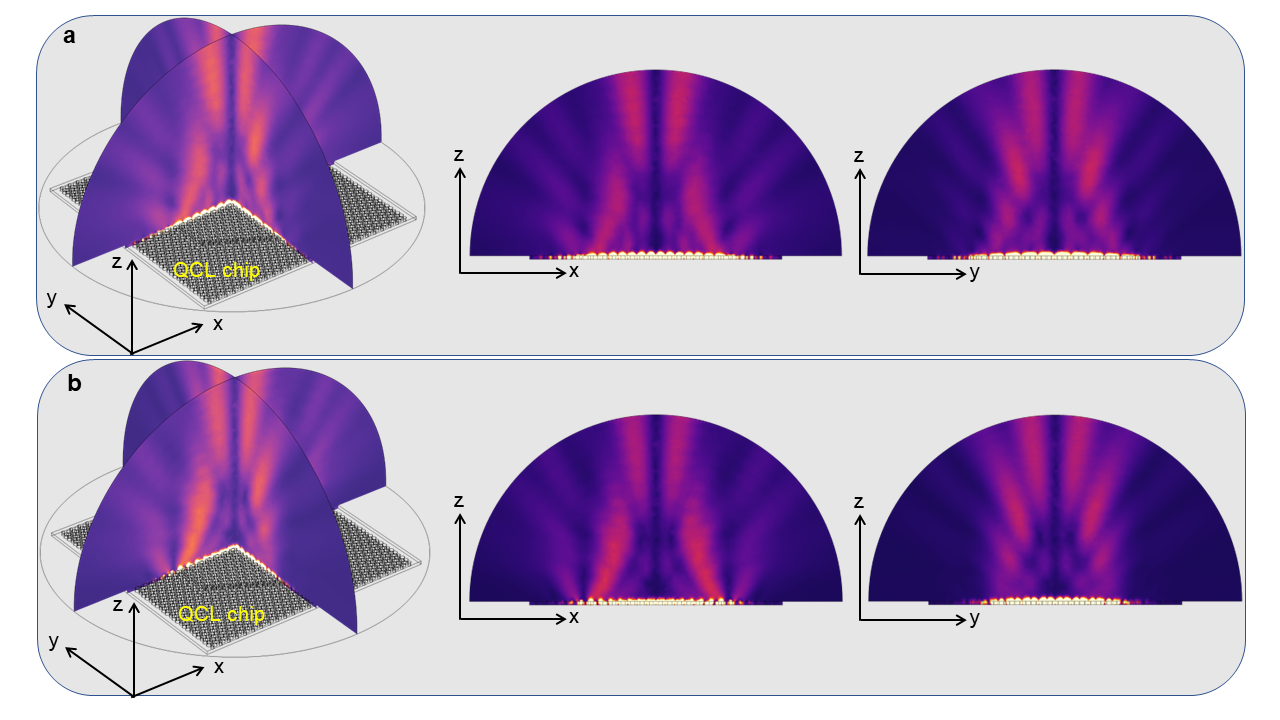


**Figure S6**. 3D simulation of passive laser device with the size (*L*) of the nontrivial cavity with *N* =15. Even the experimental measurements only show one lasing mode, the simulations always present two degenerated modes, as shown in **a** and **b**, respectively.


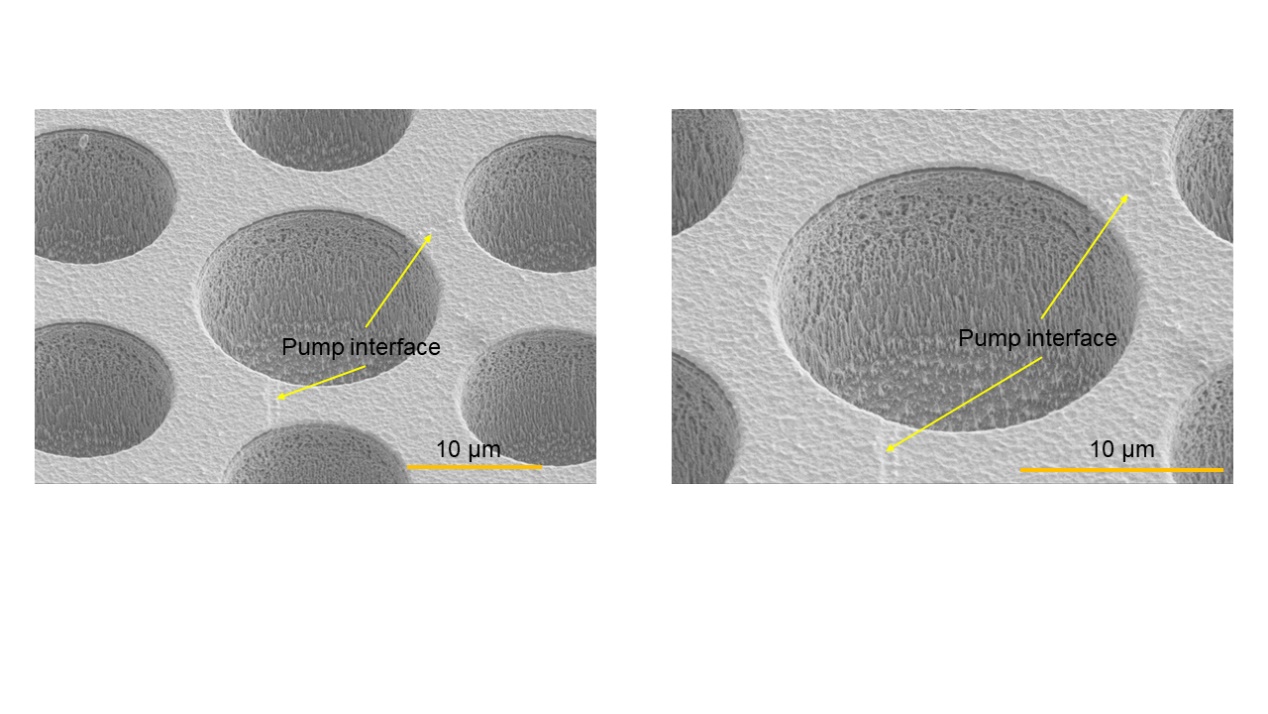


**Figure S7**. The zoomed-in SEM photos at the pump interface. Such perspective view to show the etched side wall of the air holes. Moreover, a clear step is observed for the pump boundary, where the higher side is isolated by SiO_2_ layer with thickness of 250 nm, and the pump side is lower with the Au/Ti film directly contacting with the QCL.


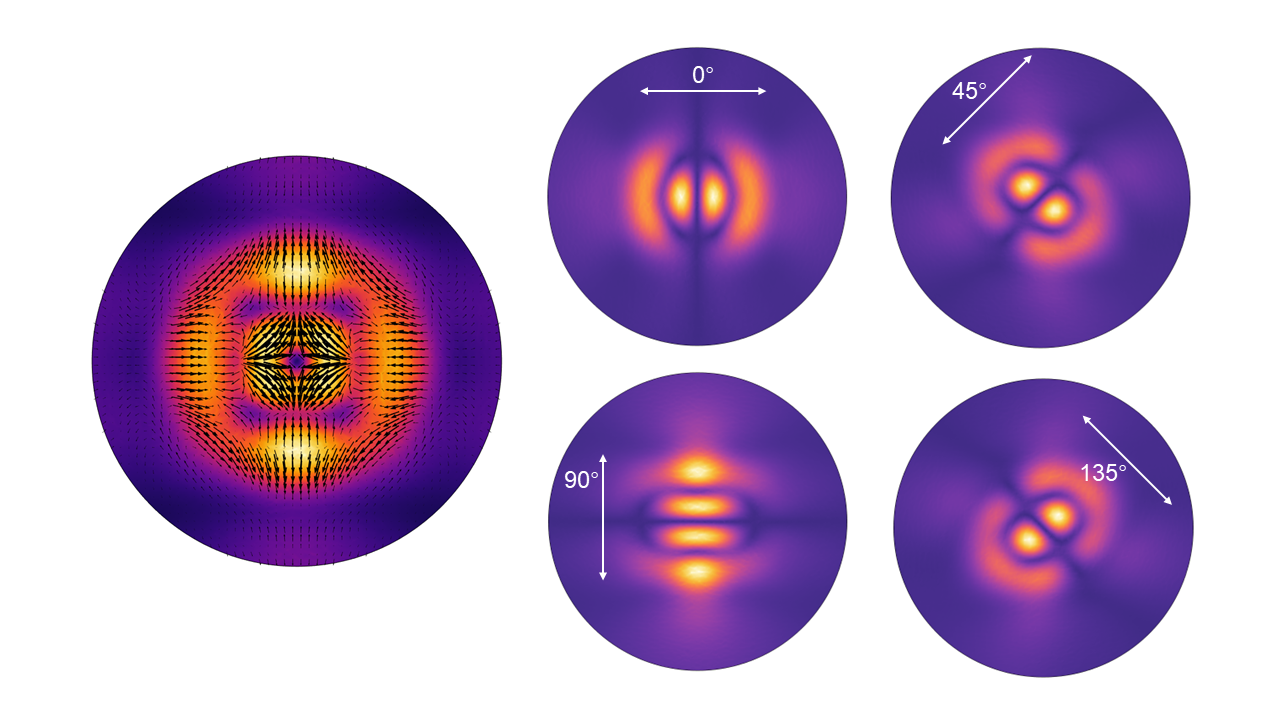


**Figure S8**. Numerically calculated far-field beam profile for the device N=7 and the polarization-resolved beams from 0° to 135°.


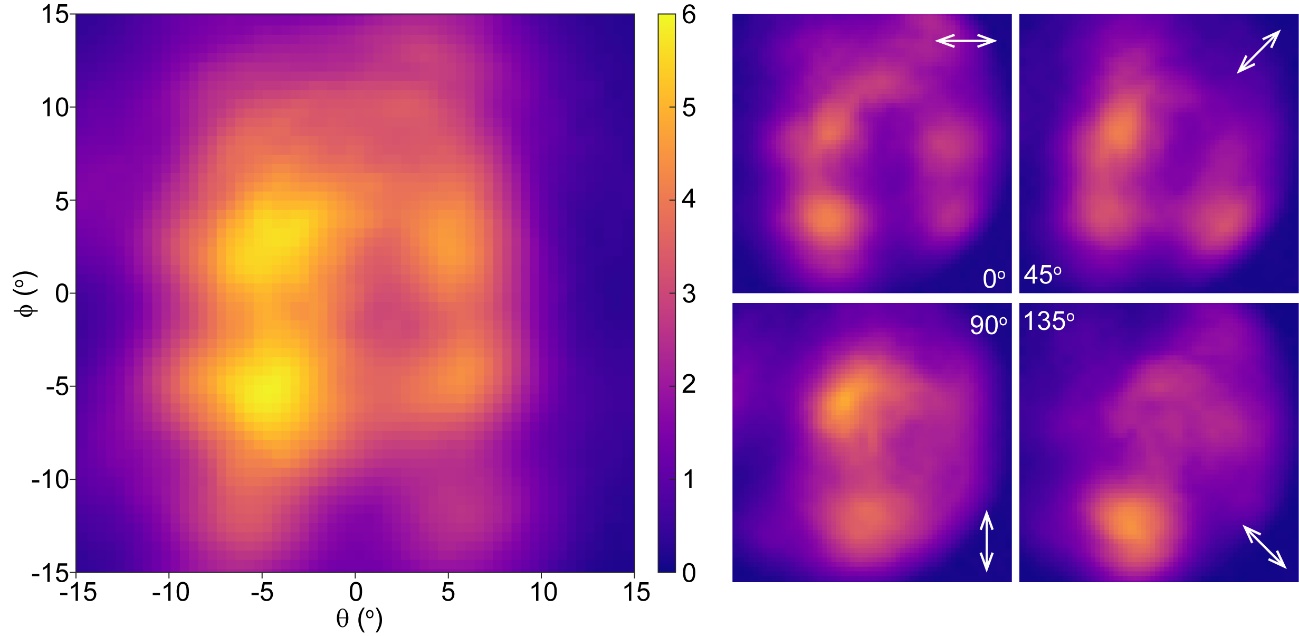


**Figure S9**. Experimentally measured far-field beam profile for the device N=7 and the polarization-resolved beams from 0° to 135°.


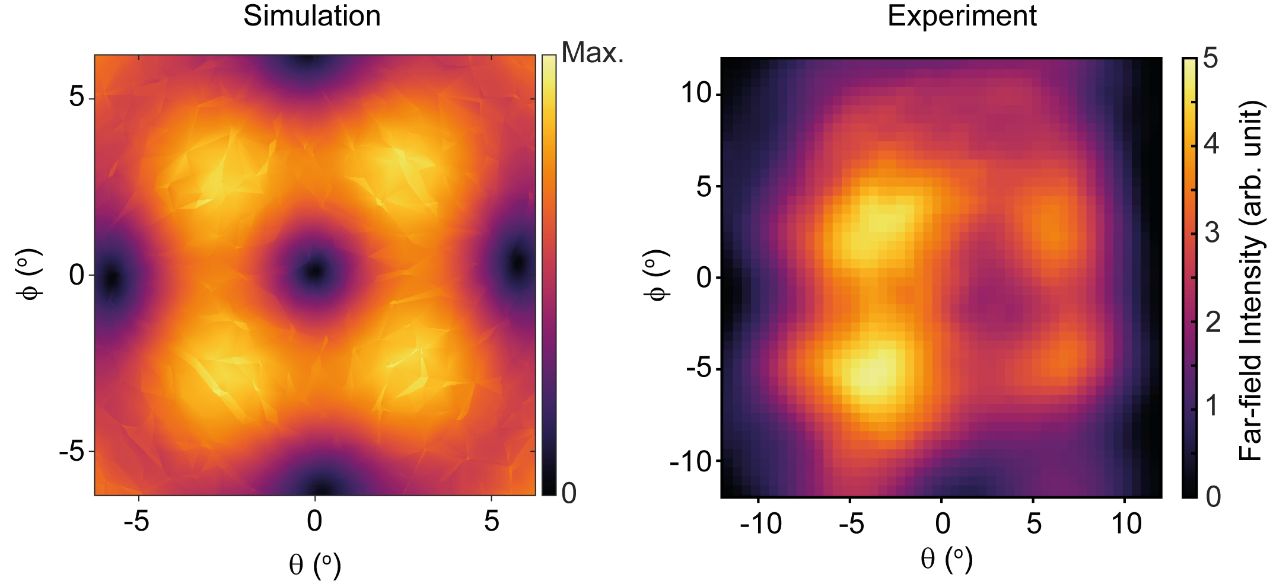


**Figure S10**. Comparison of numerically simulated and experimentally measured far-field beam profiles. For the simulation, only the contribution from the topological nontrivial domain (i.e., bulk BIC) is taken into consideration. The laser device with photonic lattice constant of *a* = 35 μm, and the nontrivial domain has N = 7.


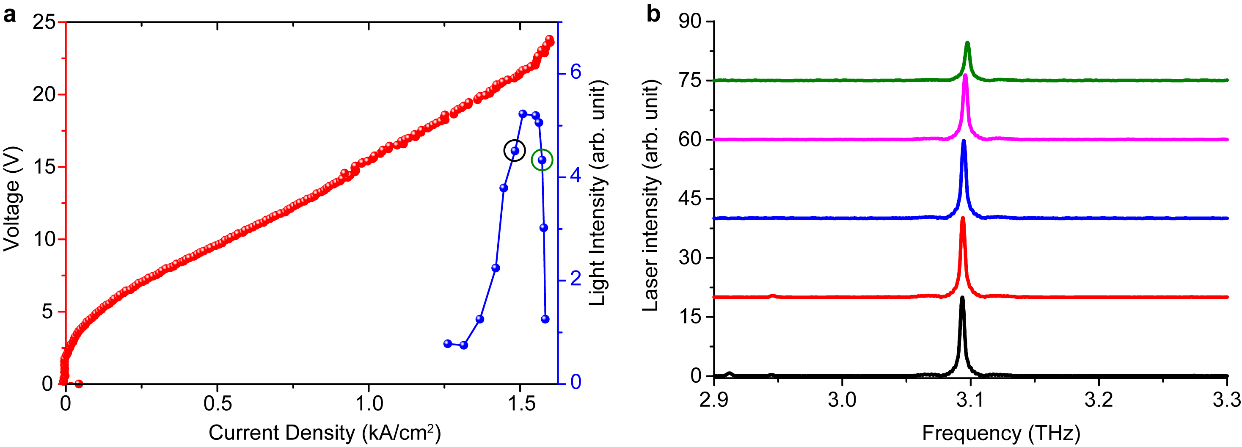


**Figure S11**. **a**, The L-I-V curves and corresponding laser spectra from the pre-rollover, rollover, and after-rollover. The laser device with photonic lattice constant of *a* = 34 μm, and the nontrivial domain has N = 11. **b**, The lasing mode appears at the tail (high frequency) of gain spectra. As results, the laser intensity is relative lower compared to the laser device with N = 7 (in main text, *a* = 35 μm,) even the pumping area is larger. However, a single-mode lasing peak is observed that is driven by the topological bulk BIC mode.
